# Recent advances in probiotication of fruit and vegetable juices

**DOI:** 10.5455/javar.2023.j706

**Published:** 2023-09-30

**Authors:** Md. Saydar Rahman, Dwip Das Emon, Maria Afroz Toma, Asmaul Husna Nupur, Poly Karmoker, Abdullah Iqbal, Mohammad Gulzarul Aziz, Md. Abdul Alim

**Affiliations:** Department of Food Technology and Rural Industries, Bangladesh Agricultural University, Mymensingh, Bangladesh

**Keywords:** Probiotication, fruit juice, vegetable juice

## Abstract

Probiotics are live bacteria beneficial to health when consumed adequately. Health professionals now recommend probiotics on regular diets due to their positive effects on human health. The probiotics that are usually consumed from the market through food products are mostly dairy-based. Fruit and vegetables are gaining popularity as preferred matrices for probiotic carriers to the human body, owing to their high cholesterol content and the lactose intolerance of dairy products. On the other hand, fruits and vegetable juices are rich in nutrient content such as vitamins, minerals, and antioxidants and do not contain a starter culture that can compete with the nutrients. The probiotication of fruit and vegetable juices (apple, carrot, citrus fruit, pome­granate, watermelon, tomato, and pineapple) are performing as efficient probiotic bacteria carriers. This review covers the previous works that highlighted the variety of probiotic fruit and vegetable juices as well as the viability of each probiotic in various products after proper fermentation and storage. In addition, physicochemical and sensory changes that occurred during the processing and storage period have been discussed. Furthermore, strategies (microencapsulation, adding prebiotics, antioxidant addition, maintaining optimum pH, temperature, adaptation with resistance, and good packaging) to improve the stability of probiotic bacteria are outlined, as it is difficult to maintain the stability of probiotic bacteria during storage. Finally, the manuscript discusses the effect of probiotic fruit and vegetable juices on human health.

## Introduction

Currently, people are paying more attention to their diets and overall wellness. For that reason, functional foods, especially probiotic-rich foods, are gaining more interest. Probiotic foods are foods fermented by probiotic microorganisms and comprise a substantial category of functional foods [1]. The International Scientific Association for Probiotics and Prebiotics states that probiotics comprise living bacteria and if administered to a host in the correct amount, benefit human health [2,3]. Probiotics are beneficial microorganisms primarily present in dairy products and provide advantages to human health [4]. One should consume no less than 10^6^ –10^7^ colony forming unit (CFU)/ml of probiotics daily to benefit from them [5,6]. Another research for probiotic ingestion recommended a minimum dose of 10^9^ CFU per serving of food products [7].

Probiotics generally contain various types of bacteria, for instance, *Lactobacillus*,* Streptococcus*,* Lactococcus*,* Enterococcus*, and* Bifidobacterium* [4,8]. They do so many positive changes in our body, including increasing the operative mode of the intestinal mucosa’s barrier, balancing intestinal bacteria, reducing inflammation, promoting the growth of helpful bacteria, soothing gastrointestinal discomfort, stopping diarrhea, and restricting the spread of pathogens, among other health advantages [9,10]. Probiotic bacteria are also used to combat aspergilli and reduce the availability of aflatoxin and other food pollutants [11].

Juices made from fruit and vegetables are considered a good substitute, especially given that their market value is expanding at the quickest rate globally [12]. Previous studies [13–15] identified probiotic strains acceptable in dairy goods, for example, cheese, yogurt, dairy drinks, and ice cream. Customer preferences for healthier substitutes have changed due to the growing knowledge of milk’s allergenicity and high amounts of cholesterol and fat [16]. Although it has seemed that dairy products were the primary source of probiotic bacteria until now, the popularity of fruit and vegetable juices is increasing more rapidly [16]. Fruit and vegetables include polyphenol compounds, which are good providers of vitamins and minerals and have a very appealing profile for people of all ages [17–19]. Some probiotic lactic acid bacteria (LAB) can ferment slightly acidic plant and vegetable-derived substances, making them a potential probiotic source [20,21]. The nutritional components of fruit and vegetables are moderators of metabolic simulators and detoxifiers of carcinogens [22].

The idea of paraprobiotics and postbiotics in probiotic diets is essential and currently understudied. Paraprobiotics or nonviable microbes benefit consumer health [23,24], and postbiotics are metabolic by-products produced by live bacteria beneficial to the host [4] and are released during or after lysis. Postbiotics are thought to play a role in immune system regulation and maintaining the balance of gut microorganisms, even if the exact mechanism through which they exert their effects is not entirely understood. When live microorganisms cannot be employed, such as in food preservation or people with weakened immune systems, they may be used as an alternative to live probiotics.

Probiotics can be challenging to incorporate into juice products since it can be difficult to keep the bacteria alive throughout processing and storage and ensure they get to the gut alive. Also, the behavior of probiotic bacteria is influenced by acidity, high osmotic pressure, salt concentration, heat treatment, metabolic products, dissolved oxygen, and strong redox potential while stored [25]. On the other hand, probiotic bacteria may impact a product’s flavor and fragrance, particularly [26]. The prebiotic effect of flavonoids, betacyanins, and phenolic acids, which promote the growth of probiotic bacteria, has just recently been discovered by a few research studies [27,28]. The bioaccessibility and utility of phenolic chemicals can be enhanced by general microbial metabolic processes in fruit-derived matrices [27].

The review’s objective was to provide an overview of current research on probiotics in fruits and vegetable juices as well as recommendations for ways to increase the probiotic bacteria in these liquids, including the use of prebiotics [29], vitamins, modified atmospheric packaging, using polyphenol and flavonoid-rich extracts [30], microencapsulation [31], and maintaining storage temperature [32].

## Probiotic Fruit Juices

Fruits and vegetable-rich diets are linked to the safeguarding and postponement of age-related problems and a lower incidence of many chronic diseases [33,34]. Many fruit juices are now successfully probioticated, including those from citrus fruits and others. According to a study by Zhu et al. [35], *Lactobacillus sanfrancisscensis* flourished in apple, tomato, and orange juices and reached 6–7 log CFU/ml after storage at 4^°^C. The main class of dietary polyphenols in citrus fruits is known as citrus flavonoids. Bioflavonoids are being considered more in cancer treatment due to their high bioactivity and strong antioxidant effects [36]. Healthy bacteria, such as *Lactobacillus *and* Bifidobacterium* genus, impart physiological effects that are advantageous to the host when they ferment nondigestible oligosaccharides acquired from citrus fruit by-products, such as lemon peel [37,38] and are incorporated into the concept of functional foods. Table 1 exhibits a range of probiotic fruit juices and the persistence of each probiotic in various products after adequate fermentation and storage.

The characteristics of the food matrix, different microbes, and their interactions have impact how viable probiotics are. Probiotic bacteria gradually lose viability in fruit juices while they are being stored. It might result from fermentation, lowering pH, and increasing acidity [39]. According to a study, the counts of *Lactobacillus acidophilus *and* Lactobacillus fermentum* in samples of grape juice decreased by 7.7% and 7.6%, respectively, from the 14th to 21st day of storage kept at 4°C 40]. Similarly, after the 21st day of storage, the ability to survive *Lactobacillus delbrueckii*,* Lactobacillus rhamnosus*, and *Lactobacillus plantarum* in grape juice substantially decreased [35]. In a study by Hossain et al. [41], two probiotic *Lactobacillus* strains, *L. fermentum* and *L. plantarum* were introduced in star fruit and apple juices to produce probiotic juice. After 21 days of refrigeration storage, both strains in fermented star fruit and apple juices decreased their viability. The viability of both lactobacilli in apple juice samples varies from 10^6^ to 10^8^ CFU/100 ml. In star fruit juice, the viability was 10^6^ CFU/ml until day 14, when cell counts began to decline [41]. Several examples are shown in Table 1.

Introducing prebiotics, maintaining the ideal storage temperature, microencapsulation, and other techniques increased the probiotic bacteria’s capacity to survive in fruit juices. For guava juice stored in a refrigerator, the amount of *L. acidophilus* dropped to 6.4 from 7.0 log CFU/ml when the sample was treated with 2% inulin [42]. When a probiotic sample is stored in the refrigerator, *L. rhamnosus* increases to 7.7 from 6.9 log CFU/ml [42]. Deshpande et al. [43] found that the colony count of probiotic bacteria cultures was reduced from 3.0 × 10^9^ to 1.5 × 10^9^ when *Lactobacillus bulgaricus *and* L. plantarum *were encapsulated in sweet orange juice. Another study found that free-living probiotics in apple and orange juice decreased rapidly following the 4 weeks of refrigeration storage and could not survive after 5 weeks. In contrast, encapsulated bacteria survived 6 weeks [44].

**Table 1.** The variety of probiotic fruit juices and the persistence of each probiotic in various products after proper fermentation and storage.

Fruits	Probiotic bacteria	Viability during fermentation/storage	Reference
Mango juice	*Pediococcus* *pentosaceus*, *Pediococcus* *acidilactici*	At 4°C, the *P. pentosaceus* sample’s viability ranged from 0.47 × 10^7^ to 2.34 × 10^7^ CFU/ml, while the *P. acidilactici* sample’s viability ranged from 0.96 × 10^7^ to 3.37 × 10^7^ CFU/ml after 4 weeks of storage. At 25°C, *P. pentosaceus* samples had a viability range of 0.34 × 10^7^–2.81 × 10^7^ CFU/ml and *P. acidilactici* samples had a viability range of 0.77 × 10^7^–4.05 × 10^7^ CFU/ml after 4 weeks of storage.	[32]
Mango + carrot juice	*L. rhamnosus*, *L. acidophilus*, *L. plantarum*	*L. plantarum*, comparable to *L. rhamnosus*, demonstrated maximum viability after 24 h of fermentation. After 35 days, *L. plantarum* had the highest average count, 8.56 log CFU/ml, followed by *L. rhamnosus*, 8.35 log CFU/ml, and *L. acidophilus* had the lowest, 7.49 log CFU/ml.	[45]
Mango beverage (MRS broth + 10% fruit Juiice)	*L. paracasei*, *L. casei*, *L. rhamnosus*	No discernible difference between *L. casei *and* L. paracasei*. In the mango drink, *L. paracasei* was more resilient than *L. rhamnosus*.	[46]
Mango juice	Abt-5 culture: *Bifidobacterium*, *L. acidophilus*, *S. thermophilus*, Fiti-culture: *L. rhamnosus* Yo-mix culture: *L. bulgaricus* *S. thermophilus*	After 48 h of fermentation, Fiti culture: 6.73–9.14 log CFU/ml Yo-mix culture: 5.25–8.2 log CFU/ml Abt-5 culture: 5.4 log CFU/ml	[47]
Guava juice	*L. rhamnosus*, *L. acidophilus*	After refrigerating storage, in the control sample, the *L. acidophilus* number decreased to 6.4 log CFU/ml from 7.0 log CFU/ml. When samples are treated with 2% inulin, the *L. acidophilus* number decreases to 6.9 log CFU/ml from 7.0 log CFU/ml. *L. rhamnosus* count increases to 7.7 log CFU/ml from 6.9 log CFU/ml in control. When a sample is treated with 2% inulin, the *L. rhamnosus* count decreases to 6.8 log CFU/ml from 6.9 log CFU/ml.	[42]
Grape juice	*L. fermentum*, *L. acidophilus*	The initial readings were 7.61 log CFU/ml for *L. acidophilus* and 7.0 log CFU/ml for *L. fermentum*, respectively. On day 14 of storage, both probiotic counts increased maximum.	[40]
Sweet orange juice	*L. bulgaricus*, *L. plantarum*	The viability of probiotic LAB cultures was changed from 3.0 × 10^9^ to 1.5 × 10^9^ with encapsulated probiotic cells.	[43]
Orange juice	*Lactococcus lactis*	For the extrusion method of microencapsulating *L. lactis*, seven different herbal-based hydrogels were combined with varying prebiotic concentrations. All gel formulations demonstrated excellent viability stability during 6 weeks of storage at 4°C in orange juice.	[48]
Orange juice	*P. acidilactici*	At 4°C and 30°C, the probiotic juice has counts between 7.2 and 8.5 log CFU/ml.	[49]
Coconut water (packaged coconut water and fresh coconut water)	*L. casei*	*Lactobacillus* concentration was between 10^8^ and 10^9^ CFU/ml at 36°C with a cultivation time of 48 h.	[50]
Coconut water + inulin	*L. rhamnosus*	There was 82 × 10^8^ CFU of probiotics per milliliter. The product in refrigeration has a 15-day shelf life.	[51]
Apple juice	*L. plantarum*	After 48 h of fermentation, *L. plantarum* had reached its maximum viability of 8.37 log CFU/ml.	[52]
Fuji apple juice	*L. casei*, *L. plantarum*, *L. acidophilus*	With notable increases in viable cell counts from 7.5 to 8.3 log CFU/ml stored at 4°C for 30 days.	[53]
Apple, orange, and tomato juices	*L. sanfrancisscensis*	At the end of storage, the probiotics in all three juice samples were still viable and exceeded the standards for probiotic food (>10^6^–10^7^ CFU/ml)	[35]
Apples, pears, and carrots beverage	*L. plantarum*	The viability was 8 log CFU/ml during the 14-day storage period at 35°C.	[54]
Apple and star fruit juice	*L. fermentum*, *L. plantarum*	*L. plantarum *decreased from 3.6 × 10^8^ to 1.7 × 10^8^, *L. fermentum* decreased from 5.6 × 10^8^ to 2.9 × 10^8, ^and mixed isolates decreased from 5.23 × 10^8^ to 2.4 × 10^8^ in probiotic apple juice during 21 days of refrigeration.	[41]
Star fruit juice	*L. rhamnosus*, *L. helveticus*, *L. paracasei*	Final cell counts after 8 h of storage at 30°C were found to be 10^8^ CFU/ml	[55]
Cherry juice	*L. rhamnosus*, *L. plantarum*, *L. paracasei*, *L. casei *	All fermented samples showed a considerable reduction in sucrose and malic acid, which underwent malolactic fermentation to become lactic acid. The fermented juices from *L. rhamnosus *and* L. paracasei* had the highest percentage of propyl acetate.	[56]
Cornelian cherry juice	*L. plantarum*	At refrigerated storage, Free cells: 7.36 log CFU/ml in the fourth week; Immobilised: 9.95 log CFU/ml	[57]
Pomegranate juice	*L. plantarum*, *L. acidophilus*	During the storage period at 4°C, *L. acidophilus* was more viable than *L. plantarum*. Within 2 weeks, viable cells remained at their highest level, but after 3 weeks, they sharply declined. *Lactobacillus acidophilus* decreased from 12.5 × 10^7^ CFU/ml to 6.9 × 10^7 ^CFU/ml after 4 weeks, but *L. plantarum* decreased from 4.2 × 10^7^ CFU/ml to 3.3 × 10^7 ^CFU/ml.	[58]
Pomegranate juice	*L. paracasei*	At 4°C for 28 days of storage, *L. paracasei* decreased from 9.2 to 7.2 log CFU/ml.	[59]
Pineapple juice	*B. lactis*, *L. plantarum*	Adding prebiotic FOSs had no impact on the probiotic bacteria’s survival capacity. The cell counts of *Bifidobacterium* were between 10^8^ and 10^9 ^CFU/ml, while Lactobacilli’s cell counts were between 10^9 ^and 10^10 ^CFU/ml.	[60]
25% Whey + 75% pineapple juice	*L. acidophilus *	At 4°C, the beverage had viable probiotic cell counts of 4.92 log CFU/ml and 4.2 log CFU/ml after 42 and 56 days, respectively.	[61]
Pineapple, raspberry, and orange juice	*L. casei*	Microcapsules viability in, Pineapple juice: 100% (2.3 × 10^7^ CFU/gm spheres); Orange juice: 91% (5.5 × 10^6^ CFU/gm spheres); Raspberry juice: Viability dropped off quickly and vanished after the storage time.	[62]
Watermelon juice	*L. paracasei*, *L. plantarum*, *L. acidophilus*	The *Lactobacillus* count in probioticated watermelon juice (2%, 5%, and 10%) showed high viability (10^8^–10^9^ CFU/ml) during fermentation at 30°C for 72 h.	[63]
Watermelon juice	*L. plantarum*	Until 2 weeks of chilled storage, the viability of *L. plantarum* with or without FOS or inulin supplementation was maintained at about 11 log CFU/ml.	[64]
Apricot juice	*L. casei*, *B. longum*, *B. lactis*, *L. acidophilus*	*B. lactis*,* B. longum*,* L. casei*, and *L. acidophilus* strains produced monoculture fermentation cell yields of 7.2, 7.25, 7.06, and 7.16 log (CFU/ml), respectively, while mixed culture fermentation produces greater yields.	[65]
Passion fruit pulp	*L. reuteri*	30°C and pH 3.18 were determined to be the ideal growing temperatures for *L. reuteri* in the passion fruit pulp. When incubated for 48 h at 30°C, 20°C, and 10°C, the viability was discovered to be 10–11 log CFU/ml.	[66]
Passion fruit juice + yam flour	*L. casei*	It was proven that passion fruit could successfully cover up the unpleasant flavor of *L. casei* fermentation. The microbe had >10^6^ CFU/ml during storage (28 days).	[67]
Barberry juice	*L. plantarum * PTCC 1058	It took 48 h for the live *L. plantarum* cell to reach 8.91 log CFU/ml, which was higher than the 7.00 log CFU/ml average during the 28-day storage period at 4°C.	[68]
Fig juice	*L. delbrueckii*, *L. casei*, *L. plantarum*	Forty-eight hours of fermentation at 30^°^C resulted in the viability of roughly 9 log CFU/ml for *L. delbrueckii*. After 4 weeks of refrigeration at 4^°^C, the viable cell counts of *L. delbrueckii* and *L. plantarum* in fermented fig juice were still 6 and 5 log CFU/ml, respectively; however, *L. casei* only persisted until the second week, dropping 9–3 log CFU/ml.	[69]
Sohiong juice	*L. plantarum*	The probiotic’s viability was 6.12 log CFU/gm after 36 days of storage.	[70]
Acerola + ciriguela juice	*L. rhamnosus*, *L. plantarum*, *L. casei*	Due to spray drying, two decimal logarithm units reduced the number of viable cells. However, probiotic cell counts in all powders exceeded the minimum necessary for probiotic foods (10^6 ^CFU/ml).	[71]

## Probiotic Vegetable Juices

Vegetable juices with probiotics are a well-linked and developing trend in the health and wellness sector. To formulate these juices, vegetables are fermented with healthy bacteria, which is claimed to provide several health advantages when consumed. Juices from vegetables are suitable for people who prefer a low-fat diet because they are high in fiber and almost fat-free [72]. Vegetables may be used as a replacement for dairy goods due to their abundance of nutrients, such as minerals, carbohydrates, vitamins, and other necessary components, that the body may employ to repair, refill, and maintain its alkaline reserve [73]. The impact of probiotic juices on physiological outcomes has been discovered in numerous investigations. One study found that daily consumption of probiotics resulted in considerable reductions in body mass index, weight, and waist size in obese individuals. The probiotics also improved inflammation markers and metabolic health [74].

A study used probiotic *L. acidophilus*-containing yacon roots to make symbiotic yacon juice. In refrigerated and room conditions, the juice’s probiotic strain viability was reasonable (10^6 ^CFU/ml) for 15 and 27 days, respectively [75]. *Lactobacillus acidophilus* and *L. plantarum *at 37^°^C, both strains that flourished in juice combinations, contained respective concentrations of around 9 and 8 log CFU/ml following 24 h of fermentation [76]. In the investigation of Champagne et al. [77], the peak cell concentration in cabbage juice was 7 log CFU/ml. These investigations demonstrated that vegetables can serve as efficient probiotic carriers. Table 2 shows a variety of probiotic vegetable juices and the persistence of each probiotic in various products after proper fermentation and storage. Like fruit juices, probiotic microorganisms in vegetable juices lose viability during storage (Table 2), and some strategies improve their stability during fermentation and storage.

## Physiochemical Changes in Probiotic Fruit and Vegetables Juices

Numerous studies have shown that probiotic fruit and vegetables utilize carbohydrates during fermentation, increasing acidity and reducing pH. Throughout fermentation, the acidity and pH rapidly reduced due to the synthesis of lactic acid by LAB. Vegetables and fruits high in sugar produce more acid than other types of food. A study used *Lactobacillus paracasei*, *L. rhamnosus*, and *Lactobacillus helveticus* to ferment star fruit juice. The largest quantities of lactic acid were produced by *L. paracasei*, which also had a pH much lower than the juice made by *L. helveticus* and *L. rhamnosus*. Most of the naturally occurring esters and aldehydes in star fruit juice fell to extremely minimal or insignificant quantities; however, varied amounts of ketones, fatty acids, and alcohols were produced. All fermentations dramatically reduced it from 3.5 to around 1.9–2.0 gm/l [55]. In another study, apricot juice was fermented with *Lactobacillus casei*,* Bifidobacterium lactis*, *L. acidophilus*, and *Bifidobacterium longum*. The pH of the juice dropped from 6.6 to between pH 4.6 and 4.9 during fermentation, and certain mixtures revealed that probiotic bacteria were proliferating and metabolically active [65]. The probiotic’s fermentative action on the products during storage at 8°C for 35 days in mango and mixed carrot juice treated with *L. plantarum*, *L. rhamnosus*, and *L. acidophilus* likely contributed to the pH decreasing and acidity increasing in all samples during storage [45]. Moreover, fermented apple juice can be developed using the *L. acidophilus*,* L. casei*, and* L. plantarum* strains. These LAB strains demonstrated good growth in the apple juice throughout 72 h of fermentation with noticeable increases in viable cell counts, and lactic acid concentrations to 4.2 gm/l from 0, and a pH to 3.8 from 5.5 [53].

The total soluble solids in probiotic fruit and vegetable juices are reduced during fermentation and storage. A study showed that after 15 and 27 days of storage, the total soluble solid in symbiotic yacon juice containing *L. acidophilus* reduced from 7.8 to 5.3 and 5.5, while the acidity rose from 0.067 to 1.02 and 0.80 [75]. By adding 1% *L. plantarum*, 1% *L. fermentum*, and a combination of 0.5% each to apple and star fruit juices, three probiotic juice samples were produced and demonstrated that with longer storage times, the pH, protein content, and total soluble solids of probiotic juices all slightly decreased [41]. According to the scientist Lu et al. [55], star fruit juice fermented with *L. paracasei*, *L. helveticus*, and *L. rhamnosus* resulted in fructose levels rising from 20.5 to 22.8 gm/l on day 4 to 29.4 to 30.4 gm/l on day 8 of all fermentations. The Brix of all three probiotic strains decreased slightly from 7.09 to roughly 6.93–6.00 [55]. A different result was also found in some studies. In a combination of juice consisting of mango and carrot treated with *L. plantarum*,* L. rhamnosus*, and *L. acidophilus* for 35 days at 8°C, there was no alteration in color or total soluble solids of the products [45].

According to certain studies, fruit and vegetable juice fermented with probiotic bacteria had increased antioxidant activity and overall phenolic content. Nguyen et al. [60] found that pineapple juice fermented with *Bifidobacterium* and *Lactobacillus* strains has a small increase in total phenolic content during fermentation and decreases somewhat after storage. Another study showed that the fermentation of pomegranate juice with *L. plantarum* for 24 h and then kept for 4 weeks contained more desirable volatile components than nonfermented juice even after 4 weeks of cold storage [90]. Researchers found that the *in vitro* antibacterial and antioxidant effects of fermented apple juice were substantially enhanced by the organic and phenolic acid metabolisms. In one investigation, *Streptococcus thermophilus*, *L. fermentum*, and *L. plantarum* were used to ferment blueberry juice. Total quercetin-3-rhamnoside, rutin, ferulic acid, and phenolic concentrations of mixed fermented samples were 98.59%, 79.08%, 15.22%, and 82.19% higher after 48 h of fermentation than in the unfermented juice [91]. A different result was found when apple juice was fermented with *L. acidophilus*, *L. casei*, and *L. plantarum* showed a significant increase in total amino acids after 30 days at 4°C, but a decrease in total phenolic content and viable cell counts [53].

**Table 2.** The variety of probiotic vegetable juices and the persistence of each probiotic in various products after proper fermentation and storage.

Vegetables	Probiotic bacteria	Viability during fermentation/storage	Reference
Yacon juice	*L. acidophilus*	The LAB counts of refrigerated probiotic yacon and normal probiotic yacon decreased from 8.59 to 5.97 and 5.95 log CFU/ml after 15 and 27 days of storage, respectively.	[75]
Yacon beverage	*B. lactis*	Viability in the nonfermented yacon beverage decreased from 7.94 to 6.85 log CFU/ml after 28 days of storage. The amount of CFU in the fermented yacon beverage was only 5.09 log CFU/ml.	[78]
Cucumber-bottle gourd juice	*L. acidophilus*	At 4°C, viable counts reduce from 8.12 to 5.37 log CFU/ml.	[79]
Beetroot juice	*L. rhamnosus*,* L. plantarum*, *L. delbrueckii*	The pH and sugar content gradually decreased over time. The probiotic drink had higher total phenols, flavonoids, and antioxidant activity than the fresh juice sample. The viability was above 10^6^ CFU/ml after fermentation.	[80]
Tomato juice	*Limosilactobacillus reuteri*	After 4 weeks of storage, 5.70 log CFU/ml of viable probiotics were added to tomato juice from untreated microorganisms in free form and 5.94 log CFU/ml from sonicated cells in free form.	[81]
Tomato, carrot, and beetroot juice	*L. plantarum* *L. delbrueckii*	Following a 48-h fermentation, *L. plantarum* produced 9.27, 9.04, and 9.23 log CFU/ml in tomato juice, beetroot juice, and carrot juice, respectively, while *L. delbrueckii* produced 8.19, 8.46, and 8.09 log CFU/ml in tomato juice, carrot juice, and beetroot juice, respectively.	[72]
Tomato juice + 3% sea buckthorn juice	*L. casei*	After 16 days of storage at 4°C and 15°C, the viable cell count of *L. casei* was 9.3 and 9.4 log CFU/ml, respectively.	[82]
Pumpkin juice	*L. casei*	In just 24 h, probiotic growth reaches about 10^10^ CFU/ml. After 13 days in the refrigerator, the culture was still above 10^6^ CFU/ml.	[83]
Soymilk (soymilk yogurt)	*B. longum*,* L. plantarum*,* L. acidophilus*,* Lactococcus thermophilus*, and *L. casei*	Probiotic cultures had a more significant impact on Gram-negative pathogenesis than Gram-positive pathogenesis. Probiotic cultures were more effective against *Shigella shigae* As2, *Shigella typhimirium* As3, and *E. coli* O15H7.	[84]
Soymilk	*L. plantarum*, *P. pentosaceus*	Even after 28 days of refrigeration, both probiotic isolates’ viability was greater than 7 log CFU/ml. Additionally, they demonstrated more significant than 6 log CFU/ml viability when subjected to gastrointestinal stimulation.	[85]
Cabbage juice	*L. casei*	In the first 24 h of fermentation at 37°C, the culture increased 6.4 log cycles, reaching 10^10^ CFU/ml, before growth decreased and the stationary phase began. Throughout 14 days of storage at 4°C, the viable count maintained above 10^7^ CFU/ml.	[86]
Turnip juice + banana juice	*Bifidobacterium bifidum*,* L. acidophilus*, *L. delbrueckii*	22.5 log CFU/ml was the initial probiotic concentration. After a 14-day fermentation, the probiotic concentration grew to 28 log CFU/ml over the length of storage.	[87]
Cucumber juice	*L. acidophilus*	Microbial viability in cucumber juice containing 3% prebiotic was 3.2 × 10^10^ CFU/ml. Microbial viability in the juice with 2% stevia was 1.9 × 10^8^ CFU/ml. The juice's microbiological viability was 2.1 × 10^8^ CFU/ml at 3% inoculum size.	[88]
Carrot, tomato, and purple cabbage juice	*L. plantarum*	*L. plantarum* cells rose from 7.49 to 9.13 log CFU/ml after 48 h of fermentation at 30°C.	[89]
Jicama, winter melon, and carrot	*L. acidophilus *and* L. plantarum*	Inoculated with *L. acidophilus* and *L. plantarum*, after 24 h of 37°C fermentation, both bacteria multiplied in the juice combinations, reaching almost 9 and 8 log CFU/ml, respectively. At the end of storage, *L. plantarum* viability was 4.57 log CFU/ml.	[76]

## Sensory Changes in Probiotic Fruits and Vegetable Juice

Numerous studies have supported the sensory acceptance of probiotic fruit and vegetable juice. Because several metabolites or organic acids are generated throughout processing, manufacturing, and storage, sensory changes after including a probiotic strain may affect the product’s odor, flavor, color, and texture [92]. Researchers used *Lactobacillus reuteri* to ferment apple juice in a study to explore the effects of LAB fermentation on the aroma and functional composition of apple juice. This result illustrated the positive impacts of fermentation on juice by altering the nature and concentration of volatile compounds, primarily esters and alcohols, in fermented juice [93].

Many research investigations have examined the sensory quality of probiotic vegetable and fruit juice decrease with increased storage durations. According to research by Hossain et al. [41], the sensory assessment of apple and star fruit juices fermented with *L. fermentum* and *L. plantarum* and control samples steadily decreased with extended storage times. Juices from winter melon, jicama, and carrot incubated with *L. acidophilus* and *L. plantarum*, the degradation of total carotenoids varied from 12% to 23% during fermentation periods and 16% to 23% during cold storage [76]. Acevedo-Martinez et al. [46] showed that the acceptability of the mango beverage, which had undergone fermentation by *L. rhamonus*,* L. casei*, and* L. paracasei*, went from a value of 7 at initial days to 6 by 1 week, and stayed stable till the end of the 4th week in storage.

Specific authors discovered methods to enhance the sensory changes in probiotic fruit and vegetable juices. According to Deshpande et al. [43], probiotic sweet orange juice with encapsulated and free strains had an overall acceptability rating of 8.3 and 7.8 in the first week. After 4 weeks of storage, juice that had been encapsulated outperformed than juice that had been free, scoring 7.5 versus 7.0 overall. According to Do and Fan’s [76] investigation, enhancing the item’s sensory acceptance by adding sucrose or multifruit juice was successful. Combining the two beverage categories can increase the variety of beverages available while improving the final product’s flavor [94].

## Strategies to Improve the Stability of Probiotic Bacteria

The strain, the nature of the juice, the additional form, and the ingredients have an impact on the probiotic’s survival [81]. By choosing the appropriate strain and juice type, probiotic bacteria in juices could be kept more stable. Fruit drinks can add probiotic cultures as biomass or straight freeze-dried cultures [95]. Novel strategies for adding probiotics to foods have been investigated to address these problems. Probiotic bacteria can improve juice quality by producing some lactic acid, which changes the juice's flavor and chemical composition. Microencapsulation is a promising method for protecting probiotic microorganisms from hazardous conditions. There are numerous methods that scientists are now using to increase the probiotic bacteria's viability in fruit and vegetable juices.

### Prebiotic inclusion

Prebiotics are nondigestible substances that encourage the development of healthy bacteria in the gut. They contain indigestible carbohydrates, for instance, dietary fibers, as well as resistant starches. According to recent research works, adding probiotics to fruit and vegetable juices can increase the probiotic bacteria’s survivability. The gut microbiota’s fermentation metabolites are best modulated by prebiotics [96]. Eating products with prebiotics and probiotics helps balance the gut flora, prevents the growth of pathogens, and improves overall health, as claimed by Balthazar et al. [97].

With the help of prebiotics, probiotics can withstand the challenging conditions of the gastrointestinal tract since prebiotics act as the source of energy and nutrients for the probiotics. For instance, in a study, orange juice with dextran and oligosaccharides was evaluated in a fermented system with fecal bacteria after *in vitro* digestion. Results indicated that the gut flora’s composition was improved by orange juice [98]. In another study by Zoghi et al. [99], adding 2.5% inulin to the samples in synbiotic apple juice had a noticeable impact on *L. acidophilus* viability. In a different study, *L. casei* and *L. acidophilus* were microencapsulated in alginate beads coated with chitosan by adding inulin and galacto oligosaccharides. The viability test was performed in orange juice. For *L. acidophilus* and *L. casei*, the proportions of cells with galacto-oligosaccharides were roughly 0.4 and 0.5 logs greater than those without galacto-oligosaccharides [100].

Prebiotics’ impact on the development of *L. casei* in two different media, De Man, Rogosa, and Sharpe (MRS) broth and mango drink, was examined in a study. Cell density in MRS and MRS + 5% sucrose was higher than in the absence of prebiotics after 24 and 48 h, respectively. However, adding inulin and fructo-oligosaccharides (FOSs) showed favorable benefits toward the end of the 54 h test period, and the prebiotic treatments and controls had significantly different outcomes. The addition of 5% FOSs during the 48 h incubation period markedly increased the development of *L. casei* in the mango beverage [46]. According to Freitas et al. [29], FOSs and sucrose boost *L. casei* viability in fermented acai beverages. Adding 5% inulin increases the activity of *Lactobacillus* spp. against *Escherichia coli* and their survival ability [101]. Hesam et al. [102] discovered that the viability of *Bifidobacterium animalis* spp. *lactis* in unfermented pomegranate juice was increased by xylo oligosaccharides compared to not using it. The symbiotic beverage’s metabolic activity was measured by a noteworthy pH drop, including a rise in titratable acidity.

### Microencapsulation

The technique of coating tiny droplets of active ingredients with a tiny capsule is known as microencapsulation [103]. Microencapsulation has been discovered as a prospective approach to increase the viability of probiotic bacteria in fruit and vegetable juices. Probiotic viability is only affected by how they are produced, stored, and digested, but the microencapsulation method can shield them from hostile surroundings [104]. Probiotic bacteria, however, are susceptible to environmental conditions such as temperature, pH, and oxygen, which can significantly diminish their viability and efficacy when taken in liquid form. The probiotic microorganism is an example of a material that can be protected by being microencapsulated into tiny particles or droplets. This coating can offer physical and chemical protection against the harsh elements in fruit and vegetable juices, such as their acidic pH and high temperatures [105]. In addition, probiotics can be slowly released using microencapsulation, enabling more efficient delivery to the stomach [103]. Probiotic microorganisms can be contained in a protective matrix via spray drying, extrusion, or emulsions [106].

Studies have explored using microencapsulation to increase the probiotic bacteria’s viability in fruit and vegetable juices. According to a study by Naga Sivudu et al. [107], after 6 weeks of storage in refrigerated conditions, probiotic bacteria and yeast microencapsulated cells in carrot and tomato juices lose viability at a lower rate than free cells. Another study states *Bifidobacterium adolescentis* cells enclosed in an emulsion had higher survival rates than free cells [108]. Furthermore, the physical characteristics of microcapsules and the cell count were studied about various core-to-wall ratios and formulations of the wall material. The sample with a core-to-wall 1:1 ratio had a considerable advantage over those with a core-to-wall ratio of 1:1.5 in bulk density, encapsulation efficiency, and cell number [104]. In another study, to increase bacterial survivability in orange juice with low-pH and gastrointestinal situations, researchers developed *L. rhamnosus* microbeads using alginate and a mixture of alginate and xanthan gum. Compared to alginate microbeads, gum-coated alginate microbeads demonstrated better encapsulation efficiency [109].

A study used the complicated coacervation process to make microcapsules containing *L. acidophilus*. These microcapsules were then cross-linked with transglutaminase and added to various fruit juice. *Lactobacillus acidophilus* was protected by the microcapsules, ensuring the probiotic’s survival [110]. Different encapsulating techniques have been found. When compared to alternative encapsulating techniques, the extrusion approach is straightforward and inexpensive [31]. A minimum viability loss of 2 logarithm units was seen following spray-dried microencapsulation of acerola and ciriguela fruit juice with *L. rhamnosus*, *L. plantarum*, and *L. casei*. The logarithmic CFU/gm values for probiotic powdered drinks range from 8.00 to 8.53. This finding suggests that the probiotic cells may be somewhat securely contained using this method [71]. A study looked at how adding organic polymeric and colloidal filters affected the ability of *L. casei* inside calcium alginate microgels. The outcomes demonstrated that probiotic viability was enhanced by the addition of polymeric or colloidal fillers to the microgels when the gastrointestinal tract was stimulated [111].

Even after 4 weeks of storage, the healthy probiotic sweet orange juice with encapsulated strains of *L. bulgaricus* and *L. plantarum* maintains an excellent viable cell count (10^6^ CFU/ml) [43]. In another study, apple juice microencapsulated with *Lactiplantibacillus plantarum* coated with a core-to-wall ratio of 1:1.5 had much higher viability than those coated with a core-to-wall ratio of 1:1 by maltodextrin and resistant starch [112]. A probiotic juice that has been sodium-alginate microencapsulated is more stable, particularly at 20°C [81]. According to a study by Gandomi et al. [113], *L. rhamnosus* microencapsulated with chitosan alginate showed a 4.5 times higher survival rate after preservation in apple juice for 90 days than the probiotic in free form. Raspberry, pineapple, and orange juice were exposed to *Lacticaseibacillus casei* in the experiment of Olivares et al. [62]. Two probiotic supplements—one in free form and the other in sodium alginate-encapsulated form—were examined for each drink. The retrieved microcapsules for pineapple and orange juice had 100% and 91% viability after 28 days of cold storage at 4°C. The viability vanished with raspberry juice in its place. The occurrence was presumably brought on by the high anthocyanin absorption rate discovered inside the microcapsules. In another study, isolates of whey protein and FOSs were utilized in the experiment employing freeze-dried banana powder to act as a protective layer and maintain the survivability of two probiotic strains, *L. acidophilus* and *L. casei*. After being stored at 4°C for more than 30 days and microencapsulated, the product became more bacterially stable [114]. Giordano et al. [81] have demonstrated that sodium-alginate microencapsulation produces a probiotic juice that is more stable, particularly at 20°C. Thus, microencapsulation of probiotic bacteria may be a unique option to incorporate probiotics in nondairy products such as fruit and vegetable juices, especially those that contain low sugars and harsh environments for probiotics to grow.

### Maintaining optimum pH and temperature

By maintaining ideal pH and temperature levels, the viability of probiotics in fruit and vegetable juices can be improved. One factor that influences the viability of probiotics is the pH of the liquid. The type of fruit or vegetable used, the fermentation process, and other factors might affect a juice's pH. Probiotics are sensitive to pH variations, and extreme pH values can harm or kill them [115]. Therefore, keeping a juice's pH level within a range that's good for probiotics is crucial. Utilizing pH-adjusting substances, such as citric acid or sodium citrate, is one method for keeping a juice's pH stable. These substances can assist in bringing a juice's pH down to a level more conducive to probiotics. In addition, probiotics that can tolerate acid may be advantageous because these strains are more resilient to pH shifts. Rafiq et al. [116] found the ideal fermentation pH for *L. plantarum*,* L. casei*,* L. acidophilus*, and *B. longum* is 6, while the ideal fermentation temperature is 30°C. In another study, *Citrus limetta* was fermented with *L. plantarum*; pH 3.4 and 37°C provided the best conditions for fermentation [117].

Temperature is another element that impacts the probiotic’s survivability in juices. Probiotics are sensitive to temperature changes and susceptible to injury or death [115]. Therefore, keeping a juice's temperature within a range that's good for probiotics is crucial. Temperature-controlled storage and transportation are methods for preserving a juice's temperature. This can assist in preventing the juice from being subjected to high temperatures that might destroy the probiotics. The use of thermostable probiotics, which are more resistant to temperature changes, may be advantageous. The impact of various storage methods on a fermented food product was examined in a study. The findings indicated that freezing or cooling for over three months would be the ideal storage condition to sustain a load of live microorganisms [118]. According to Lai et al.’s [119] assessment of the viability of *L. rhamnosus* in hawthorn berry tea, the free probiotic revealed a viability loss of 77.3% and 44.8% after 4 weeks of storage at 25°C and 4°C, respectively. At storage temperatures of 4°C and 37°C, Perricone et al. [120] assessed the amount of time required to eliminate 1 log CFU/ml of *L. reuteri* in orange, apple, and pineapple juices. They discovered a notable drop in the latter temperature.

### Adaptation with resistance

The probiotic’s capacity to adjust to the circumstances in fruit and vegetable juices is one of the most critical factors in their growth and survival. The existence of other microbes, temperature, and parameters such as pH are included in this. Their capacity to withstand various types of stress is another crucial aspect of probiotics’ potential to survive in fruit and vegetable juices. This encompasses chemical and physical stress, such as that caused by preservatives and other chemicals, and physical stress, such as heat and shear. DNA repair, accumulation of suitable solutes, metabolic pathways of lipid modification, proteases and chaperones, and reactive oxygen species detoxification are the critical adaptive processes bacteria trigger to deal with such damage [121,122].

Probiotic bacteria need to be exposed to sub-lethal conditions for a brief time to induce resistance and adaptation before being pumped into the food matrix [123]. Mustafa et al. [124] investigated the effects of pH 2.5, 4.0, and 5.5 on the viability of *L. casei* in pomegranate juice and found that the pH variation hurt the probiotic’s growth, especially at pH 2.5 and 5.5. They indicated that a feasible strategy to reduce cell death and damage while processing, storing, and transiting through the gastrointestinal tract is to subject probiotics to acid stress before mixing them into fruit juices and then storing them in the refrigerator.

### Packaging

Juices made from fruit and vegetables may contain less viable probiotics due to several variables, including temperature, pH, and the presence of other bacteria. A good package can solve this issue. Cabello-Olmo et al. [118] examined how different storage methods affected fermented products. They discovered that standard packaging positively impacted the microorganisms' viability. Pimentel et al. [125] looked at how the packing methods affected the viability of *Lacticaseibacillus paracasei* in apple juice. They found that glass containers maintained probiotic viability better than plastic ones. What matters most is how much less oxygen-permeable glass is than polyethylene. As per a study on packaging techniques for enhancing probiotic viability in fruit and vegetable juices, these methods may help boost the survival of the bacteria in these products.

### High-intensity ultrasound (HIUS)

The term “ultrasound” refers to the waves of sound that are significantly higher in frequency(>16–20 kHz) beyond the range of human audibility. HIUS is defined as ultrasonic waves with intensities greater than 1 W/cm^2^ and frequencies between 20 and 500 kHz [126,127]. The impact of ultrasonic technology on microorganisms is linked to DNA damage, hotspot creation, free radical production, and the rupturing and shearing of cell wall membranes [128]. Food composition, probiotic culture type, and process variables, including frequency, processing time, power, pulse mode, and duration, significantly impact the HIUS’s effects [129]. It is a potentially valuable and intriguing technology that can boost probiotic bacteria’s activity and survival. Applying ultrasonic inhibited and accelerated probiotic cell growth and viability [130].

**Figure 1.**
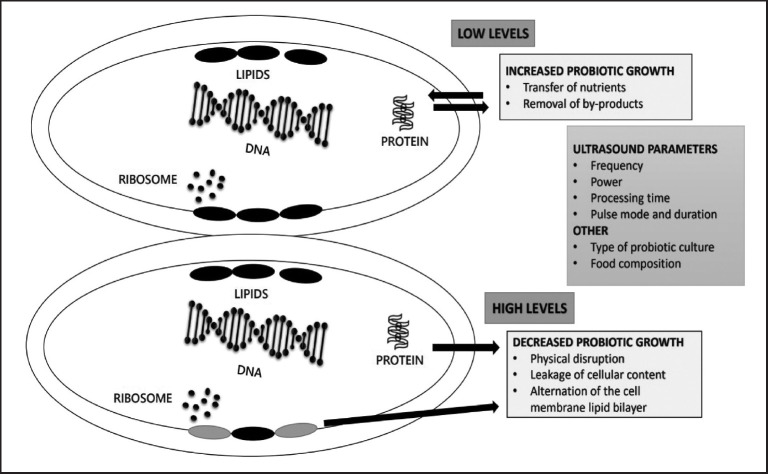
Impact of HIUS on probiotic growth based on sonoporation levels [129].

Figure 1 shows how HIUS affects probiotic growth dependent on sonoporation level. Sonoporation refers to the temporary creation of holes in a cell membrane due to sonication. By improving the permeability of cell membranes, which permits for increased mass transfer of substrate across the membranes and effective elimination of waste products from cellular metabolism, microbial viability can be enhanced with ultrasound at low levels of sonoporation. As a result, microorganisms receive oxygen and nutrients more quickly. Due to the physical breakage and alteration of the lipid bilayer that makes up the cell membrane, high levels of sonoporation can result in cell death and leakage of cellular material [131].

The viability of the probiotics for 28 days of storage at 7°C was examined in a study employing HIUS for processing durations of 2.5, 5, 7.5, and 10 min in the probiotic strawberry drink. The findings demonstrated that HIUS accelerates microbial viability and shortens the fermentation process in cultured probiotic strawberry drinks [132].

## Impact of Probiotic Fruits and Vegetable Juice on Human Health

Although yogurt and other dairy products are frequently linked to probiotics, a growing trend focuses on adding probiotics to fruit and vegetable juices. A safe substitute for dairy probiotics is the probiotication of plant-based juices to produce functional foods. They stimulate consumption for those who are lactose intolerant, are devoid of cholesterol, and lack several dairy allergies [16]. These beverages’ probiotic additions include advantages such as improved digestive health, a boosted immune system, and a higher nutritional profile. People can maximize the benefits of these beneficial bacteria and strive toward optimum health and well-being by including probiotic fruits and vegetable juices in a balanced diet. In addition to increasing the nutritional value of fruits and vegetables, forming various probiotic fermented fruit and vegetable juices using fermentation technology also organically integrates probiotics and their metabolites with prebiotics, promoting intestinal health, and preventing and treating chronic diseases [133].

Among other health benefits, probiotics can reduce allergic reactions and infectious diarrhea, regulate immune cells, and soothe gastritis. Probiotics contribute to the partial balance of the gut microbiota, the complex community of bacteria in our gut. Fruit and vegetable juices high in probiotics can help with digestive problems, including bloating, gases, and constipation [134,135]. Clinical trials on probiotics have been conducted for several purposes, including preventing the development of antibiotic-assisted diarrhea and for the development of several conditions, including irritable bowel syndrome, prevention of allergies, vaginitis, *Helicobacter pylori* infection, and necrotizing enterocolitis in infants [136]. Gut microbiota can be modulated by the active ingredients in foods fermented by probiotics [137]. It may change the gut microbiota’s interaction with the host’s biological functions, such as producing inflammatory response mediators, vagal neurotransmission, neurotransmitter activity, and neuroactive metabolites [138]. In the study by Park et al. [9], a probiotic formula containing both *L. acidophilus* and *B. longum* was administered orally to infants admitted with rotavirus illness. A placebo (probiotic-free skim milk) was also given to the infants. Following the completion of the program, the probiotic group’s diarrhea lasted noticeably less time than the placebo groups did. The probiotic dosage improved symptoms such as nausea and vomiting, fever duration, and diarrhea.

A robust immune system has also been related to probiotics. The gut is critical for immune activity since many of human immune cells live there. People who drink probiotic-rich fruit and vegetable juice may be able to lower their risk of illness and improve their general immune system. In a study, in a mouse model of poly-sensitization, mucosal administration of both *B. longum* and *L. paracasei* during sensitization and challenge resulted in a significant down-regulation of allergen-specific immune responses and inhibition of airway inflammation [139]. Probiotic bacteria can interact with and activate commensal microflora and intestinal immune cells to influence specific immune functions and immunological homeostasis [140]. Lu et al. [141] showed that *B. animalis* sup F1-7 might function as a potent activator to module the immunological response by supporting the expression of caspase 8/3, the Bid and Bak genes and proteins as well as by activating the FAS/CD95 pathway. In the study by Miri et al. [142], the expression of cell junctions was influenced by the strains of *Lactobacillus* and *Bifidobacterium*, which may have modulated homeostasis.

## Challenges in the Probiotication of Fruits and Vegetable Juice

The advantages of probiotics primarily depend on how much of them are present in the foods and how well they endure difficult digestive circumstances. A key element of probiotication is the number of probiotics present in fruit and vegetable juices at the time of ingestion. Juices may not be helpful for probiotics as a growth medium because some substances may affect them negatively. The type of probiotics used is another issue in the probiotication of fruit and vegetables. A particular juice promotes the growth of probiotics. Finding an excellent probiotic strain for a specific juice is difficult. Probiotics in fruit and vegetables also struggle because of sensory changes, substrate shortages that impede the growth of probiotic species with higher requirements, and stress brought on by unit operations during processing [31]. Juice made from fruit and vegetables that have been probioticated faces several difficulties that must be overcome if the desired health advantages are to be achieved. More investigation is required to overcome these difficulties and guarantee the security and efficiency of juice products containing probiotics.

## Future Perspective

While food is a possible source of probiotic bacteria, there is still much to learn about how these microbes interact with food and how that impacts human health. Probiotics are necessary for controlling the immune system and gut flora, which aids in preventing diseases, including respiratory conditions. To halt the loss of probiotic culture, innovative and cost-effective methods such as microencapsulation are needed. Although the scientific community is becoming more interested in the food matrix and the process conditions, continual research is required to improve the process and understand the ideal settings due to each product’s distinct qualities. To encourage the use of these products as innovative probiotic choices, more research is needed.

## Conclusion

The probiotication of fruit and vegetable juices to supply the gut with bacteria that promote health has gained popularity in recent years. The article illustrated the most recent findings in probiotic fruit and vegetable juices. Juices made from probiotic fruit and vegetables have been demonstrated to provide a range of advantages, including enhancing immune function, lowering the risk of certain diseases, and improving gastrointestinal health. Probiotic-infused fruit and vegetable juices will significantly benefit lactose-intolerant people who refrain from consuming dairy items. Fruit and vegetable juices are effective probiotic carriers. Numerous studies have shown that the juice of numerous fruit and vegetables can efficiently transport probiotic microbes. Future advances in this field will likely be much more fascinating as studies in this area are gaining more attention.

## List of abbreviations

CFU: colony forming unit; FOS: fructo oligosaccharides; HIUS: high-intensity ultrasound; LAB: lactic acid bacteria; MRS: De Man, Rogosa and Sharpe.
